# Clinical Characteristics and Molecular Profiling of *SF3B1*-Mutated Myelodysplastic Syndrome (MDS) in a Real-World Practice

**DOI:** 10.3390/ijms27031423

**Published:** 2026-01-30

**Authors:** Ruonan Roni Wang, Hein Than, Christopher Tham, Gee Fung How, Si Jie Khoo, Tertius T. Tuy

**Affiliations:** 1 Department of Haematology, Singapore General Hospital, Singapore 169608, Singapore; roni.wang@mohh.com.sg (R.R.W.); hein.than@singhealth.com.sg (H.T.); christopher.thamsw@mohh.com.sg (C.T.); how.gee.fung@sgh.com.sg (G.F.H.); khoo.si.jie@sgh.com.sg (S.J.K.); 2 Department of Haematology, National University Hospital, Singapore 119074, Singapore

**Keywords:** myelodysplastic syndrome, MDS, *SF3B1*, next-generation sequencing

## Abstract

*SF3B1*-mutated myelodysplastic syndrome (MDS) is a distinct entity associated with a favorable prognosis. Recent data suggest that certain *SF3B1* variants portend a worse prognosis. Our study aims to (1) describe *SF3B1*-MDS patients from a single tertiary center in Singapore and (2) determine if variant type holds prognostic value. We identified MDS patients with *SF3B1* variants via next-generation sequencing (NGS) performed from 1 November 2021 to 31 October 2025 at Singapore General Hospital. Extracted genomic material from marrow or blood samples was amplified. Libraries were prepared, sequenced, and analyzed, and the hematological parameters, mutation profiles, and outcomes were evaluated. Twenty-five patients had *SF3B1*-MDS. Ten *SF3B1* variants were found, and the three most prevalent were *K700E* (42%), *K666N* (19%), and *R625C* (7.7%). The median variant allele frequency (VAF) was 30% (IQR: 11–36%). Twelve patients (48%) had ≥1 co-mutations. Variant type and VAF had no impact on disease progression; only the presence of ≥1 co-mutations increased the progression chances. In our study, the analysis of *SF3B1* variant type was inconclusive and showed no demonstrable statistical association with disease progression. However, the number of co-mutations affected the prognosis of patients. As *SF3B1*-MDS is heterogenous, further studies are needed to capture its diversity and identify features required to improve risk stratification and personalized treatment.

## 1. Introduction

Myelodysplastic syndrome (MDS) is a heterogenous group of clonal myeloid neoplasms characterized by ineffective hematopoiesis, leading to peripheral blood cytopenia, and a variable risk of transformation to acute myeloid leukemia (AML) [1,2]. For nearly two decades, risk stratifications were heavily reliant on the Revised International Prognostic Scoring System (IPSS-R), which used the severity and degree of cytopenias, bone marrow blasts, and cytogenetic groups to prognosticate survival and evolution to AML [3]. The landscape of MDS classification and prognostication has evolved over recent years to incorporate molecular features that better capture clinical–pathological entities and predict clinical outcomes. The International Prognostic Scoring System—Molecular (IPSS-M) incorporates individual somatic gene mutations along with hematological and cytogenetic data [4]. Treatment of MDS is often risk-adapted, varying from supportive care to higher-intensity therapies, including hypomethylating agents (HMAs) and allogeneic hematopoietic stem cell transplant (HSCT).

Splicing factor 3b subunit 1 gene, *SF3B1*, encodes for U2 small nuclear ribonucleoprotein and is one of the core splicing factors; it is crucial for proper spliceosome complex formation required for appropriate RNA splicing. Mutations in *SF3B1* have been associated with a diversity of cancers, both non-hematological and hematological [5,6]. In particular, it has been associated with MDS and is recognized as one of the most frequently mutated genes in MDS, detected in up to a third of all MDS patients [5,7]. *SF3B1* mutation has been linked to the presence of ringed sideroblasts (RSs) and erythroid dysfunction in the bone marrow, with reported mutation rates reaching up to 65% in those with RSs [8]. The latest World Health Organization (WHO) classification recognizes *SF3B1*-mutated MDS as a distinct MDS entity [4].

However, the presence of mutated *SF3B1* is generally associated with a favorable outcome and an indolent disease course for MDS [5]. However, emerging data may suggest that not all *SF3B1* mutations are the same, with certain *SF3B1* variants, such as *E592K* and *K666N*, portending a worse prognosis [1,2,9,10]. Furthermore, certain co-mutations like *ASXL1* are associated with a worse prognosis than having either mutation in isolation, while other co-mutations like *TET2* may conversely have better prognoses than having either alone [11,12]. Lastly, de novo AML patients with *SF3B1*, as well as other mutations like *SRSF2*, *USAF1*, *ZRSR2*, *ASXL1*, *EZH2*, *BCOR*, and *STAG2*, comprise a cohort of patients known as secondary AML patients and fair clinically worse [13].

In our study, we look at the incidence of *SF3B1* variants in MDS patients in a single tertiary institution and report their clinical characteristics and disease outcomes.

## 2. Results

A total of 25 MDS patients had *SF3B1* mutations identified by NGS. Baseline demographics, hematological and bone marrow parameters, risk strata, and *SF3B1* indices are listed in Table 1. Patients were stratified by the presence or absence of *K700E* and K666* (K666* includes the various *K666 SF3B1* substitutions). The *SF3B1*-mutated MDS patients included 14 men and 11 women with a median age of 72 years (IQR: 68, 78). Hemoglobin levels and absolute neutrophil counts (ANCs) were similar when stratified between K666* and *K700E* status. Only platelet counts showed a difference in both strata; K666* SF3B1 MDS patients tend to have a lower platelet count on diagnosis with 343 (IQR: 144, 378) versus 100 (IQR: 22, 193), *p* = 0.007. In contrast, patients with *K700E* variant MDS presented a higher platelet count of 368 (IQR: 144,443), while non-*K700E* patients had a median platelet count of 167 (IQR: 89, 286), *p* = 0.03. Thirteen of the twenty-five patients had <5% blasts on the initial bone marrow aspirate, and eleven out of twenty-five had ≥5% or more RSs. For the entire population, the median percentage of RSs in the marrow was 1% (IQR: 0%, 30%). When stratified according to K666* and *K700E* status, the median amounts of RSs were not statistically significant. For non-K666* versus K666*, the median amounts of RSs were 28% (IQR: 0%, 48%) versus 0% (IQR: 0%, 2%), with *p* = 0.07. Similarly, the median amount of RSs for *K700E* was 28% (IQR: 0%, 54%), and for non-*K700E* it was 1% (IQR: 0%, 18%), with *p* = 0.4. Cytogenetic subgroups included normal cytogenetics, complex karyotype, monosomy 7, and trisomy 8 (+8); these cytogenetic features were incorporated into the IPSS-M risk stratification. No instances of deletion 5q (del 5q) were observed in this group. Patients with *K700E* tend to have “Low” or “Very Low”-risk IPSS-R scores compared to non-*K700E* patients. The distribution of IPSS-R scores was similar when stratified by K666* status. Patients with a higher number of co-mutations tend to have less favorable IPSS-M scores. On further analysis, 7 out of the 25 *SF3B1*-mutated cases (28%) fell into the IPSS-M high-/very high-risk categories.

### 2.1. SF3B1 Variant, Clonality, and Co-Mutations

**Figure 1:**
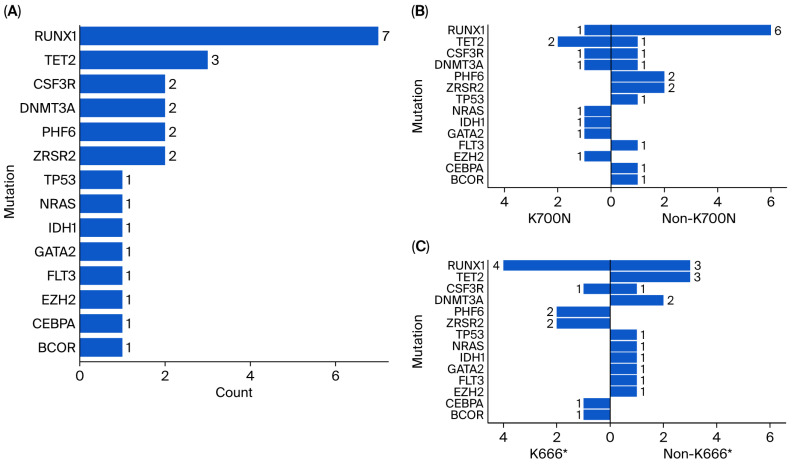
Co-mutations found in *SF3B1*-MDS patients. (**A**) Bar chart of all the different co-mutations found in the entire cohort; (**B**) co-mutations stratified by *K700E* and non-*K700E* status; (**C**) co-mutations stratified by K600* and non-K600* status.

In Table 1, the median *SF3B1* VAF was 30% (IQR: 11%, 36%), with no difference between the K666*/non-K666* and *K700E*/non-*K700E* strata. The breakdown of co-mutations is shown in Figure 1. The co-mutations found were *BCOR*, *CEBPA*, *EZH2*, *FLT3*, *GATA2*, *IDH1*, *NRAS*, *TP53*, *ZRSR2*, *PHF6*, *DNMT3A*, *CSF3R*, *TET2*, and *RUNX1*, with *RUNX1* being the most prevalent.

Ten variants of *SF3B1* were found (Table 2), with the top three most prevalent being *K700E* (42%), *K666N* (19%), and *R625C* (7.7%). One patient had two different *SF3B1* variants: *K700E* and *T663I*. The median VAF was 30% (IQR: 11–36%). All the mutations were missense mutations. Twelve out of twenty-five (44%) patients had one or more co-mutations.

### 2.2. Therapy and Outcomes

Eleven out of the twenty-five patients received treatment for MDS; among them, seven received HMA, two were recruited into the MBG453 trial (TIM3 inhibitor), two received intensive chemotherapy, and three went on to receive allogeneic HSCT. At a median follow-up of 20.3 months, out of the twenty-five patients, ten patients progressed, four due to worsening cytopenias, four due to increasing bone marrow blasts > 10%, and two due to disease progression to AML. Only one patient had died at the time of analysis.

There were 11 patients with *K700E*. Five patients had *K666N*, and two patients had *R625C*. In a multivariate analysis (Table 3), none of the *SF3B1* variant types were associated with different outcomes. When grouped together as high-risk *SF3B1* variants, *K666N* and *R625C* did not have an inferior progression compared to those without the high-risk *SF3B1* variant. Similarly, when comparing the K666* to the non-K666* variants, there was no difference in progression, with an HR of 1.66 (95% CI: 0.47–4.92; *p* = 0.4). Lastly, having *K700E* did not demonstrate a difference in disease progression compared to non-*K700E* variants, with an HR of 0.29 (95% CI: 0.06–1.38; *p* = 0.12). Male gender led to an HR of 5.30 (95% CI: 1.04–26.9; *p* = 0.044). In a similar manner, the number of co-mutations was associated with an increased chance of progression, with each additional co-mutation resulting in increasing chances of progression (HR of 2.78, with 95% CI: 1.5–5.16; *p* = 0.001).

## 3. Discussion

### 3.1. Co-Mutations (Number and Type)

In this study of *SF3B1*-mutated MDS patients, with each increasing co-mutation, there was a 2.8-fold higher chance of disease progression requiring treatment. Disease progression was defined as a composite of worsening cytopenias, an increase in bone marrow blasts to >10%, progression to AML, or death. Only two patients progressed to AML, and given the small cohort size (n = 25), meaningful statistical analysis was not feasible for leukemic transformation alone. The co-mutations in our cohort were *BCOR*, *CEBPA*, *EZH2*, *FLT3*, *GATA2*, *IDH1*, *NRAS*, *TP53*, *ZRSR2*, *PHF6*, *DNMT3A*, *CSF3R*, *TET2*, and *RUNX1*. In the literature, *SF3B1* is associated with improved prognosis; however, studies suggest that certain co-mutations can potentially change the prognosis for *SF3B1*-mutated MDS. Though there were multiple co-mutations detected, they were not comprehensive, and the number within the cohort was far too small to determine if specific mutations combined with *SF3B1* fared worse or better than others. In contrast, the analysis by Malcovati et al. showed that in 482 *SF3B1*-mutated MDS cases with co-mutations, the number of co-mutations did not affect overall survival compared to cases without co-mutations [10]. And yet they did show that having two specific co-mutations of *RUNX1* and *EZH2* worsened the outcome [10]. Similarly, Huber et al. and Yun et al. demonstrated that *RUNX1* mutations combined with *SF3B1* mutations resulted in a 3.5-fold decreased survival [9,14]. Song et al. demonstrated that *SF3B1* and *ASXL1* had a negative prognostic factor for survival [11]. In contrast, Song et al. investigated MDS patients with *SF3B1* co-mutated with *DNMT3A* and demonstrated similar survival to isolated *SF3B1*-mutated MDS but better survival than isolated *DNMT3A*-mutated MDS [15]. Lastly, co-mutations with *TET2* did not demonstrate any significant benefit compared to having an *SF3B1* mutation alone [12]. The wide range of co-mutation combinations remains complex, and further studies are needed to elucidate their significance. Thus, it would be ideal for a larger study to ascertain if *SF3B1* combined with certain co-mutations, compared with having these specific mutations in isolation, would result in better or worse outcomes.

### 3.2. Variant Type/Hotspot

In our study, when comparing *SF3B1* variants with each other, none of them had a statistically different progression—neither the high-risk *K666N* and *R625* variants, nor the favorable *K700E* variant (*p* = 0.12). It is possible that with the increase in our cohort numbers, the positive prognosis associated with the presence of *K700E* may become more evident. Likewise, due to the low numbers of *SF3B1*-MDS patients (N = 25), with only seven having *K666N* (n = 5) or *R625C* (n = 2) variants, these variants’ effects on prognosis may not have been sufficiently forthcoming. There are an increasing number of studies that suggest that not all *SF3B1*-mutated MDS should be assessed as one single group, as the prognostic impact varies between the different variants. In many studies, the most frequently mutated *SF3B1* variants at *K700E* account for 48–60% of *SF3B1*-mutated MDS, which is associated with the typical RS-MDS and overall improved prognosis [5,16,17,18,19]. Mutations at the *K700E* site led to gain-of-function mutants which confer the typical positive prognosis [18]. Conversely, though non-*K700E* variants are less common, these *SF3B1* variants not only lack the favorable prognosis but also tend to be associated with higher-risk MDS at presentation, higher rates of leukemic transformation, and worse survival [5]. Kanagal-Shamanna et al. analyzed 94 mutated *SF3B1* and 415 wild-type *SF3B1*-MDS genes and found that non-*K700E* variants had a lower ANC on presentation, a higher IPSS risk stratum, and associated *RUNX1*, *BCOR*, *IDH2*, and *SRSF2* mutations [19]. Likewise, Liu et al. assessed K666 and R625 hotspots, which demonstrated poorer prognoses than their non-K666 and non-*R625* counterparts [17]. Dalton et al. and Choi et al. demonstrated that the *K666N* and *E592K* variants have worse prognoses compared to other *SF3B1* variants [1,2]. Furthermore, one study by Sakuma et al. demonstrated that there may be a gender disposition whereby K666* variants are typically associated with males, with *K700E* being more so with females [20]. Therefore, advancement in prognostication may require integrating both *SF3B1* hotspots, as well as identifying the presence of co-mutations.

### 3.3. VAF and Clonality

Lastly, in our study, the clone size measured by the variable allelic frequency of *SF3B1* had no overall effect on prognosis in terms of progression to AML or mortality. Jiang et al. demonstrated that *SF3B1* patients with a VAF ≥ 15% had improved survival compared to those with a VAF < 15% (HR: 0.29, CI: 0.02–0.92, *p* = 0.044) [21]. Lachowiez et al. also found that, for *SF3B1* VAF < 10%, there was a marginally decreased OS compared to VAF ≥ 10% (5 vs. 6.2 years, *p* = 0.04), while there was no change in leukemia-free survival [22].

### 3.4. Response to Treatment

There are studies that suggest that *SF3B1* mutations conferred improved response to certain MDS-directed therapeutics. Idossa et al. found that *SF3B1*-mutated MDS was more likely to respond to lenalidomide (56 vs. 27%, *p* = 0.04) but did not demonstrate any difference between response to HMA and erythropoietin-stimulating agonists [23]. Luspatercept has been used in patients with *SF3B1* low-risk MDS with efficacy [24,25]. Yet there are limited studies comparing *SF3B1*-mutated versus unmutated patients. Interestingly, in a study by Consagra et al., there was no statistical significance in hematological improvement between *SF3B1*-mutated versus unmutated patients (53.8 vs. 40.1%, respectively, *p* = NS) [26]. Furthermore, neither variant type (57.7 vs. 42.3%, *p* = 0.31, *K700* vs. non-*K700*) nor VAF (52.1 vs. 47.9%, *p* = 0.11, VAF ≥ 38% vs. <38%) demonstrated any impact on hematological improvement. Consagra went on to show that certain *SF3B1* co-mutations were associated with hematological improvement. Response rates were 0% with 5q co-mutation; 53.8% with any *BCOR*, *BCORL1*, *NRAS*, *RUNX1*, *SRSF2*, or *STAG2* co-mutation; and 55.8% with any other co-mutations (*p* = 0.046) [26]. Unfortunately, our study lacked the numbers to sufficiently look at response rates by type of co-mutations.

Even when *SF3B1* mutation status is known, real-world management of MDS remains individualized, due to the disease’s biological and clinical heterogeneity. Patients tend to be older, and therapeutics are frequently constrained by age-related factors, including frailty and comorbid conditions. Furthermore, treatment-related complications like iron overload from chronic transfusions can negatively affect survival. Thus, an integrative approach incorporating *SF3B1* and clinical status may allow for more precise patient-centered care.

### 3.5. Limitations

This is a retrospective study with a low sample size, which could have led to low statistical power and increased risk of type II errors. True differences in progression may have gone undetected. Furthermore, the limited sample size may have predisposed to selection and sampling bias, as our study may not have sufficiently captured the diversity of *SF3B1* mutations. Finally, stratification and multivariate analyses are limited.

## 4. Materials and Methods

### 4.1. Patients and Samples

We retrospectively identified MDS patients with *SF3B1* mutations via next-generation sequencing (NGS) performed on bone marrow aspirate samples from 1 November 2021 to 31 October 2025 in the Singapore General Hospital registry. Extracted genomic DNA and RNA were amplified, and library preparation was performed with the Oncomine Myeloid Research assay (Themo Fisher Scientific, Waltham, MA, USA) to interrogate 40 DNA genes and 29 fusion driver genes. NGS libraries were sequenced on the Ion GeneStudio S5 System (Thermo Fisher Scientific, Waltham, MA, USA) and analyzed using in-house bioinformatics pipelines and the Ion Reporter^TM^ software, Version 5.20. The patient demographics, hematological parameters, and disease outcomes were analyzed. The *SF3B1* variants, variant allele frequency (VAF), and number and type of co-mutations in each patient were analyzed. Risk stratification was performed using both IPSS-R and IPSS-M.

### 4.2. Statistical Analysis

Statistical analyses were conducted using R (version 2025.05.01). Patient characteristics, hematological and bone marrow indices, diagnostic risk strata, and *SRFB1* parameters were analyzed. Results were reported as total numbers with percentages and medians with interquartile ranges (IQRs). Categorical variables between groups were analyzed with Fisher’s exact test, and nonparametric variables had their medians calculated and compared via the Wilcoxon ranked sum test. Disease progression was defined from the time of diagnosis until the time that the patient required treatment with a hypomethylating agent, intensive chemotherapy, and/or HSCT due to (1) worsening bone marrow blasts or (2) worsening cytopenias, or (3) until the patient transformed to AML or (4) died. Univariate Cox proportional hazards regression analysis was used to identify any association between each of the variables and progression, and this was followed by multivariate analysis. *p*-values < 0.05 were considered statistically significant, though inference was limited due to low event frequency.

## 5. Conclusions

In summary, *SF3B1*-mutant MDS is biologically distinct but has a considerable amount of heterogeneity depending on hotspot variations, clonality, and the number/type of co-mutations. Although *SF3B1*-MDS is commonly associated with an indolent course and relatively favorable outcome, variables add nuance and complexity to the prognostication of MDS. The above prompts a more integrative approach for this disease group. Reliance on the presence or absence of *SF3B1* alone may lack granularity and be an oversimplification.

Nevertheless, this study is primarily descriptive and hypothesis-generating. Given the small cohort size, the prognostic significance of individual *SF3B1* variants may not have been detected. Although the number of co-mutations showed a statistically significant association with disease outcome, larger studies are required to validate its prognostic relevance and to assess co-mutational burden as an adjunct prognostic factor. Additionally, interpretation of co-mutational effects is complex, as specific co-mutations influence prognosis, as reflected in the IPSS-M risk scores. Further studies examining the interactions between *SF3B1* and other co-mutations are therefore needed to better define its prognostic impact. Finally, clinical variables in MDS (e.g., co-morbidities, transfusion dependence, iron overload, and recurrent infections) also carry prognostic value, and future larger studies should explore the interactions among clinical, cytogenetic, and molecular factors on disease prognosis.

## Figures and Tables

**Table 1:** Baseline characteristics and bone marrow parameters for *SF3B1*-mutated MDS stratified by K666* and *K700E* status.

	Overall	K666*			*K700E*		
Characteristic	N = 25 ^1^	Non-K666* N = 17 ^1^	K666* N = 8 ^1^	*p*-Value ^2,3^	Non-*K700E* N = 14 ^1^	*K700E* N = 11 ^1^	*p*-Value ^2,3^
Gender				>0.9			0.12
Male	14 (56%)	9 (53%)	5 (63%)		10 (71%)	4 (36%)	
Female	11 (44%)	8 (47%)	3 (38%)		4 (29%)	7 (64%)	
Race				0.8			>0.9
Chinese	18 (72%)	13 (76%)	5 (63%)		10 (72%)	8 (73%)	
Indian	4 (16%)	2 (12%)	2 (24%)		2 (14%)	2 (18%)	
Malay	3 (12%)	2 (12%)	1 (13%)		2 (14%)	1 (9%)	
Age	72 (68, 78)	72 (70, 78)	72 (61, 77)	0.6	73 (63, 80)	72 (70, 78)	>0.9
Hemoglobin (g/dL)	8.7 (7.6, 9.5)	8.4 (7.5, 9.5)	8.8 (8.1, 9.9)	0.5	8.7 (6.9, 9.5)	8.4 (8.0, 9.5)	0.6
ANC (×10^9^/L)	2.3 (1.4, 3.6)	2.3 (1.4, 3.6)	2.4 (1.3, 3.2)	>0.9	1.5 (1.1, 2.7)	2.8 (2.2, 3.7)	0.13
Platelets (×10^9^/L)	255 (99, 368)	343 (144, 378)	100 (22, 193)	0.007	167 (89, 286)	368 (144, 443)	0.033
Marrow Blast (%)	1.0 (1.0, 2.0)	1.0 (1.0, 2.0)	1.0 (0.5, 9.0)	>0.9	1.0 (0.0, 6.0)	1.0 (1.0, 2.0)	>0.9
Ringed Sideroblasts (%)	1 (0, 30)	28 (0, 48)	0 (0, 2)	0.073	1 (0, 18)	28 (0, 54)	0.4
Karyotype				0.7			0.12
Normal	15 (58%)	9 (50%)	6 (75%)		9 (69.2%)	6 (46%)	
-Y	3 (11.6%)	3 (16.4%)	0 (0%)		0 (0%)	3 (23.2%)	
Monosomy 7	1 (3.8%)	1 (5.6%)	0 (0%)		0 (0%)	1 (7.7%)	
del(11)	1 (3.8%)	1 (5.6%)	0 (0%)		0 (0%)	1 (7.7%)	
i(14)	1 (3.8%)	1 (5.6%)	0 (0%)		0 (0%)	1 (7.7%)	
inv(3)	1 (3.8%)	1 (5.6%)	0 (0%)		0 (0%)	1 (7.7%)	
inv(12)	1 (3.8%)	1 (5.6%)	0 (0%)		1 (7.7%)	0 (0%)	
trp(1)	1 (3.8%)	1 (5.6%)	0 (0%)		1 (7.7%)	0 (0%)	
Trisomy 8	1 (3.8%)	0 (0%)	1 (12.5%)		1 (7.7%)	0 (0%)	
der(13; 14)	1 (3.8%)	0 (0%)	1 (12.5%)		1 (7.7%)	0 (0%)	
IPSS-R				0.6			0.043
Very Low	2 (8%)	2 (12%)	0 (0%)		0 (0%)	2 (18%)	
Low	15 (60%)	10 (59%)	5 (63%)		8 (57%)	7 (64%)	
Intermediate	2 (8%)	2 (12%)	0 (0%)		1 (7.1%)	1 (9%)	
High	5 (20%)	2 (12%)	3 (37%)		5 (36%)	0 (0%)	
Very High	1 (4%)	1 (6%)	0 (0%)		0 (0%)	1 (9%)	
IPSS-M				0.3			0.5
Very Low	4 (16%)	4 (23%)	0 (0%)		1 (7%)	3 (27%)	
Low	13 (52%)	8 (47%)	5 (63%)		8 (57%)	5 (46%)	
Mod Low	1 (4%)	1 (6%)	0 (0%)		0 (0%)	1 (9%)	
High	5 (20%)	2 (12%)	3 (37%)		4 (29%)	1 (9%)	
Very High	2 (8%)	2 (12%)	0 (0%)		1 (7%)	1 (9%)	
SF3B1 (VAF%)	30 (11, 36)	33 (21, 36)	14 (5, 36)	0.2	31 (11, 39)	30 (11, 34)	0.6
No. of Co-Mutations				0.7			>0.9
0	13 (52%)	9 (53%)	4 (50%)		7 (50%)	6 (55%)	
1	4 (16%)	2 (12%)	2 (25%)		2 (14%)	2 (18%)	
2	6 (24%)	5 (29%)	1 (13%)		4 (29%)	2 (18%)	
≥3	2 (8.0%)	1 (5.9%)	1 (13%)		1 (7.1%)	1 (9.1%)	

^1^ n (%); median (Q1, Q3); ^2^ Fisher’s exact test; ^3^ Wilcoxon rank sum test.

**Table 2:** *SF3B1* variants and type/number of co-mutations.

Variant	Nucleotide Change	Protein Change	N = 26 ^1^ (%)
*K700E*	c.2098A>G	p.Lys700Glu	11 (42%)
K666*			8 (30.4%)
*K666N*	c.1998G>T, c.1998G>C	p.Lys666Asn	5 (19%)
*K666Q*	c.1996A>C	p.Lys666GIn	1 (3.8%)
*K666R*	c.1997A>G	p.Lys666Arg	1 (3.8%)
*K666T*	c.1997A>C	p.Lys666Thr	1 (3.8%)
*R625C*	c.1873C>T	p.Arg625Cys	2 (7.7%)
*D781G*	c.2342A>G	p.Asp781GIy	1 (3.8%)
*E622V*	c.1865A>T	p.Glu622Val	1 (3.8%)
*H662Q*	c.1986C>A	p.His662GIn	1 (3.8%)
*T663I*	c.1988C>T	p.Thr663Ile	1 (3.8%)

^1^ One patient had two concomitant *SF3B1* variants—*K700E* and *T663I*.

**Table 3:** Multivariate analysis for progression.

Characteristic	HR	95% CI	*p*-Value
Gender			
Female	-	-	
Male	5.30	1.04, 26.9	0.044
Age	0.94	0.89, 1.00	0.044
VAF (%)	0.97	0.92, 1.02	0.3
No. of Co-Mutation	2.78	1.50, 5.16	0.001
K666*	1.66	0.47, 5.92	0.4
*K700E*	0.29	0.06, 1.38	0.12
HR-Variant ^§^	1.23	0.26, 5.89	0.8

Abbreviations: CI = Confidence Interval; HR = Hazard Ratio; ^§^ HR-Variant defined as presence of *K666N* or *R625* variants.

## Data Availability

The data presented in this study are available on request from the corresponding author due to data privacy reasons.

## Abbreviations

The following abbreviations are used in this manuscript:

AML	Acute Myeloid Leukemia
CI	Confidence Interval
HMAs	Hypomethylating Agents
HR	Hazard Ratio
HSCT	Hematopoietic Stem Cell Transplant
IPSS-R	Revised International Prognostic Scoring System
IPSS-M	International Prognostic Scoring System—Molecular
IQR	Interquartile Range
MDS	Myelodysplastic Syndrome
NGS	Next-Generation Sequencing
RSs	Ringed Sideroblasts
*SF3B1*	Splicing Factor 3b Subunit 1
VAF	Variant Allelic Frequency
WHO	World Health Organization
